# A qualitative study of stakeholder views on the effects of provider payment on cooperation, quality of care and cost-containment in integrated stroke care

**DOI:** 10.1186/1472-6963-13-127

**Published:** 2013-04-04

**Authors:** Johanneke FMM Tummers, Augustinus JP Schrijvers, Johanna MA Visser-Meily

**Affiliations:** 1 Julius Centre for Health Sciences and Primary Care, University Medical Centre Utrecht, Utrecht, The Netherlands; 2 Rudolf Magnus Institute of Neuroscience and Centre of Excellence for Rehabilitation Medicine, University Medical Centre Utrecht and Rehabilitation Centre De Hoogstraat, Utrecht, The Netherlands

**Keywords:** Integrated care, Stroke, Payment system, Incentive, Fee-for-service, Cooperation

## Abstract

**Background:**

Stroke services are a form of integrated care which have been introduced in many countries, including the Netherlands, to improve health outcomes and processes of care by connecting the acute, rehabilitative, and chronic phases of stroke care. Limited research exists on the effects of payment systems on the functioning of integrated care services from the perspectives of those involved in providing, planning and contracting the care. This qualitative study identified stakeholder views on i) challenges in integrated stroke care associated with fee-for-service systems; ii) other possible financing models for stroke care, and iii) challenges in the implementation of an integrated financing mechanism for stroke care.

**Methods:**

Twenty-four participants were interviewed using face-to-face audio-recorded semi-structured interviews. Respondents were purposively selected from five stakeholder groups; care providers, health care managers, health insurers, experts and patient representatives. Transcribed data were coded and analysed to generate themes relating to the study aims.

**Results:**

Respondents mentioned the following challenges associated with the current fee-for-service system; inappropriate incentives for cooperation, efficiency and improving quality and the inability to exert steering power at the level of the stroke service. In addition, care is not patient-centred and the financing system is inflexible.

The respondents mentioned several solutions for the challenges, but there was no consensus amongst them. Regarding the implementation of integrated financing, respondents mentioned the following general challenges; a) the foundations of the financing system are incompatible with integrated financing, b) co-morbidity and c) the lack of evidence on the effect of integrated financing. Stroke-specific challenges were; a) the diverse patient population, b) a non-uniform care trajectory, c) unclear division of responsibility for the overall care and d) different stages of development among stroke services.

**Conclusions:**

This study provides new knowledge on stakeholder perception of the effect of payment systems and financial incentives on cooperation processes, quality of care and cost-containment in integrated stroke care. The results show that fee-for-service does not provide the right incentives for the integration of stroke care. We recommend to perform financial experiments for integrated stroke care.

## Background

The aging population, with its increase of patients in need of complex care demands a new way of organising health care [1]. The health care sector has to deal with limited resources, while costs continue to rise. This combination forces policy makers and health care planners to develop sustainable health care systems that can deal with these factors in the future [2].

To improve the content of care, there has been an increased focus on various approaches such as integrated care [3]. Integrated care is supposed to ‘glue’ the entities together, enabling it to achieve common goals and optimal results [4].

To control the total costs of health care, many strategies exist ranging from capping budgets and provider payment strategies to increased consumer payments. Despite numerous theoretical papers on this topic, no one seemed to have found the holy grail yet. Currently, payment reform is thought to have the highest potential for cost savings in health care [5] and new strategies to finance care are sought for [6-8]. While many papers have been published discussing the theoretical effects of provider payment on provider behaviour, practical evidence is lacking to a large extent. Therefore, this study was set up to provide more insight into the effects of payments systems on the quality and costs of integrated care, and to explore the opportunities of implementing other financing systems.

### Stroke care

In this study, we focus on the financing of integrated care specifically for stroke patients, because of the large costs associated with this condition, and the multidisciplinary nature of stroke care. In Europe, the average annual incidence of stroke is 141 per 100,000 for men, and 94 per 100,000 for women [9]. Stroke patients require complex multidisciplinary rehabilitation and chronic care, and the group of patients is expected to increase with the aging population.

### Stroke services

Stroke services are a form of integrated care which have been established during the last decade [10]. The aim of stroke services is to improve health outcomes and processes of care by connecting the acute, rehabilitative, and chronic phase of stroke care [11,12]. In a typical stroke service, the hospital, rehabilitation centre, nursing home and primary care providers are represented. According to a modelling study in the Netherlands, the use of stroke services compared to usual care could achieve a 13% cost-reduction [13]. It is increasingly recognized that the integration of care and cooperation between different providers can be stimulated by enabling conditions in terms of policy, legislation and financing [4,14].

The current provider payment for stroke care in the Netherlands is a fragmented fee-for-service system; every provider receives separate reimbursement for the care activities delivered. Providers who form integrated pathways are still reimbursed using the fee-for-service system.

### Research aim and relevance

The aim of this study is to answer the following research questions a) what do stakeholders identify as challenges in integrated stroke care associated with fee-for-service systems; b) what do stakeholders present as possible solutions, and c) what do stakeholders identify as challenges in the implementation of an integrated financing mechanism.

## Methods

### Research design

A qualitative research design was chosen in order to obtain a full range of authentic participant views. Interviews were chosen as the method for data collection as this is a suitable qualitative method to collect individual experiences, perceptions and opinions of respondents.

### Sampling

To achieve diversity of opinion, a purposive sampling approach was used to recruit participants from each of five stakeholder groups; care providers, health service managers, health insurers, experts and the patient organisation. More details about the definition of the five stakeholder groups is provided in the list below:

**Care providers (n=4):** clinicians with detailed knowledge about the activities of at least one stroke service, and working in the hospital, rehabilitation centre or nursing home. Individuals in this group included rehabilitation specialists, neurologists and an elderly care physician.

**Health service managers (n=5):** senior managers from institutions that had clinicians involved in stroke services. Individuals in this group included local health service executives such as stroke care-coordinators, a general manager of a care institution, a medical director of a rehabilitation centre and a program manager of the hospital.

**Health insurers (n=4):** senior employees from health insurance companies involved in contracting care services for secondary and primary care as well as integrated care specifically.

**Experts (n=9):** policy makers and senior researchers who worked in national institutions or universities with a relationship to integrated care. Examples of these institutions are those with a nation-wide strategic role in health policy and planning, healthcare financing, quality improvement, patient safety initiatives or development of clinical guidelines and protocols.

**Patient representatives (n=2):** representatives from the national patient organisation for stroke.

For every category, the researchers selected potential participants from three sources; 1) The Stroke Knowledge Network Netherlands, 2) authors who recently published in national or international journals, and 3) conference speakers. Potential participants were selected for their knowledge on the topic, and/or because of their status as opinion-leaders in this field.

### Recruitment

The potential participants were sent an email that explained the research aims and asked for their participation. In case the contacted person considered a colleague more knowledgeable about the topic, this person was contacted instead. Additional participants were selected one at a time to ensure maximum variation. Twenty-four out of twenty-six (92%) contacted individuals consented to participate. The self-reported reason for non-participation by the two other participants, a care provider and an expert, was a lack of time to participate and insufficient expertise.

### Data collection

Twenty semi-structured face-to-face interviews with in total twenty-four respondents (16 interviews with one respondent and 4 with two respondents) using a topic guide (included as Appendix) were conducted in Dutch by one researcher (JT) at the interviewee’s place of work. Related documents, provided by the respondents or publicly available, were also checked for relevant information and provided input for the interview topic list. The interviewer had an academic background and was not acquainted with the respondents prior to the interview.

The interviews were of 60 minutes duration and were audio-recorded. Participants were asked for their views on the influence of the current payment systems on the functioning of stroke services and if they experienced problems associated with the current payment system. Participants were also asked to provide their opinions on how current problems could be overcome and how payment could play a role in this. The final sample-size of 24 was reached by saturation of themes, that is, no new insights were identified in the data.

### Data analysis

Interviews were anonymised and transcribed verbatim to produce transcripts of narrative text for thematic analysis. The transcripts were returned to the respondents to check whether the transcribed data correctly reflected their opinion and experiences. Following this, minor adjustments were made.

The main themes of interest, namely i) challenges associated with fee-for-service payments, ii) solutions for these challenges and iii) challenges for integrated financing provided a framework for the initial categorisation of text.

The allocation of text segments in the interviews to these three main themes was performed by all three researchers, whereby each segment was also coded as a subtheme within the three main themes. The subthemes were defined as ‘independent aspects not influenced by other subthemes’. The process of generating subthemes was inductive and iterative. The authors used seven sessions during two months to come to consensus. Discrepancies in themes between researchers were discussed until agreement was reached.

Data were also analysed with the purpose to highlight differences and convergences in responses from different groups of stakeholders. However, few differences between stakeholders were observed. Only where a difference in opinion was apparent, this is reported. Participant narratives have been used to illustrate meaning in the themes and summaries.

### Participant consent

All participants were informed of the objective of the study and that they were free to participate or withdraw from the study at any given point. Participants gave verbal consent to be interviewed and for the interviews to be audio-recorded. Interviews were anonymized to ensure that data could not be traced back to an individual respondent and confidentiality of the participants was ensured. No formal ethics approval was requested, since according to Dutch law, ethical approval is not necessary for research concerning health professionals or experts. The persons from patient organizations were interviewed as representatives of their organization and not as patients.

## Results

### Characteristics of the respondents

The twenty-four respondents consisted of nine women and fifteen men. The number of years of relevant experience of the respondents ranged from 4 to 32 years.

### Question 1: Challenges associated with fee-for-service

The respondents identified several challenges in the current organisation of integrated stroke care that were related to the fee-for-service payments. The subthemes that emerged were *incentives, steering power, patient-centred care* and *inflexibility of the financing mechanism.* Each theme is discussed below.

#### a) Incentives

All respondents mentioned that the current fee-for-service payment system provides inappropriate or contradicting incentives when it comes to integrated stroke care.

First of all, stakeholders mentioned that there is no financial reason to cooperate*.* Several stakeholders comment that the current system rewards individual acts, as opposed to collective achievements, which does not provide an incentive to cooperate.

“At this moment, on the level of the stroke service, there is no reward for an overall good result.” [manager]

“Right now there is no incentive to cooperate, and there are also no consequences if you do not cooperate.” [expert]

Second, there is a tension between the *own interest* and *shared interest* of the stakeholders involved in providing the care;

“I always say that the elastic of the individual providers is stronger than the collective one. In the end of the day, everyone first looks at their own interests.” [expert]

The focus on the organisation’s own interests seems to be closely linked to a demand from the management level to perform well;

“There is a need to focus on your own interest. Even individual wards in the hospital need to make sure they receive enough attention, to protect their right to exist.”

Third, there is no incentive to be efficient; care providers are being paid a fixed amount and there is no need felt to be more efficient than that. If certain providers want to become more efficient, the benefits are not necessarily felt directly by them.

“An empty bed is a financial risk. There is an incentive in the current system to ensure that your beds are filled with patients who are entitled to receive an additional fee. In this way, your unit has the best figures.” [care provider]

Fourth, there is no financial incentive to work on improving quality because the basis of financing is the number of services provided.

Lastly, respondents mentioned the existence of opposing incentives even within organisations between the different groups of stakeholders such as care providers and managers;

“At the level of the care providers, I think everyone finds cooperation self-evident. When you move one level up to the managers and directors, you feel much more competition. The care providers themselves are not thinking of financial incentives. It is the managers who are guilty of optimizing their own organization.” [manager]

### b) Steering power

Because of the individual payments of the institutions, stakeholders said that it is not possible to steer or exert control at the level of the stroke service. It is difficult to address common issues such as quality or quantity of care to cooperation partners. Also cooperation is thought to improve when there is centralized steering.

“If you leave the issue of cooperation to the individual organisations, then it won’t happen. You need the unit to be directed as one piece, and you cannot expect that from the individual organisations themselves.” [manager]

Several respondents mentioned that there is a need to have one steering body or person, who both financially and organisationally runs the stroke service in order to remove current obstacles.

### c) Patient-centred care

Because of the fragmented payments and demand-oriented organisation of care, some stakeholders found that also the care delivered to the patient becomes fragmented and less patient-centred. When taking care of the patient, there is less attention for the actual needs of the patient, and the focus is on the delivery of services instead:

“Providing care is not custom-made yet. Many services are being offered to the patient, while perhaps this patient doesn’t need all those services. The financing is based on the diagnosis, and not on the needs of the patient.” [insurer]

At the same time, stakeholders mention that the formation of a stroke service specifically for stroke patients might not result in optimal patient-centred care;

“Creating a specific stroke service might be attractive for some patients, however this results in the care provider focussing only on the ‘stroke’ elements. If you make it a ‘chronic care service’, then you are creating much more coherence in all the care for the patient.” [expert]

#### d) Inflexibility of the financing mechanism

Respondents said the current payment system is inflexible when it comes to innovations in care, and shifting money to other organisations within the stroke service. In addition, the separate fee-for-service financing was said to have a negative effect on the dynamic processes in the stroke service that you aim to achieve by integrating care.

“The financing mechanism should be arranged in such a way that there is money not only to structurally finance the same system, but there should be money available to see how we can constantly improve the system.” [expert]

Also, there are certain elements of the stroke service which cannot be financed using the traditional fee-for-service system;

“Overarching functions such as a chain-care coordinator are not structurally embedded in the current financing system, because it is unclear who should receive payment for this.” [manager]

To summarize, the respondents said that the current fee-for-service system provides inappropriate incentives for cooperation, efficiency and improving quality. Another challenge mentioned was the inability to exert control at the level of the stroke service because of the financing of the separate entities. In addition, the care is less patient-centred and the financing mechanism is considered inflexible.

An interesting finding was that in contrast to the experiences of the care providers, experts, insurers and managers, the representatives of the patient organisation reported that most of these issues go unnoticed by patients themselves:

“I feel that all the organisational issues are not even noticed by the patient. Of course you hear on the radio or television about issues such as waiting times, or that the care is too costly. But when you are a patient, these discussions are not relevant, and you are not even aware of them. Your first priority is to regain very basic skills, such as reaching your mouth with a fork again.” [patient representative]

### Question 2: Possible solutions for financing stroke care

To explore other ways of financing integrated stroke care, we asked the respondents whether they saw possible solutions to the issues mentioned above. The respondents elaborated upon a) several models to finance stroke care and b) entitlements to ensure reimbursement for the stroke services used.

#### a) Models for financing

**Figure 1:**
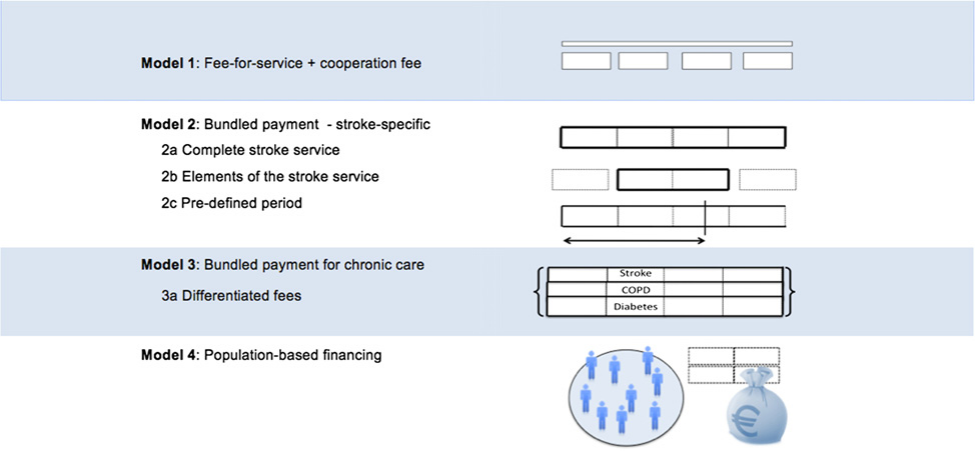
**Financing models mentioned by the respondents.** In the first model, providers receive on top of individual fee-for-service payments a joined cooperation fee. Model 2 includes three different models for bundled-payment for stroke providers. In Model 3, payment is unrelated to stroke-diagnosis and a bundled payment for all chronic care is provided. The fourth model describes population-based financing only for stroke care, or for all care delivered.

Respondents came up with ideas for different financing models (Figure 1). There was no consensus amongst the respondents regarding an optimal financing mechanism; several different options were mentioned.

In Model 1, the fee-for-service system is largely maintained, but an additional cooperation fee is added which is to cover cooperation costs. Model 2 summarizes all forms of stroke-specific bundled-payments that cover an episode of care. Respondents mentioned three sub-variants; a) covering the complete stroke service, b) covering only elements of the stroke service such as the rehabilitation phase, or the chronic phase, and c) covering all costs for a pre-defined period (e.g. 6 or 12 months). Model 3 and 4 are financing models that provide payments unrelated to stroke-diagnosis. In Model 3, a bundled payment for all chronic care is created; providers receive similar bundled payment for a patient with for example COPD, diabetes or a stroke. Model 4 refers to population-based payments. In this payment system, a pre-defined region receives a budget, based on general or disease-specific epidemiological data, and this budget should cover all care provided by the providers in that region.

Care providers in general came up with mechanisms such as Model 1 and 2, while insurers and experts generally discussed solutions resembling Model 3 and 4.

#### b) Entitlement to reimbursement

Apart from the financing model, respondents also referred to the problems with the current entitlement used in health care; the number of care activities preformed. Most respondents thought that rewarding the volume of services is an undesirable method in health care, as it leads to a focus on production instead of quality of care. Respondents mentioned to finance care-outcomes instead, as in a pay-for-performance system. Suggested entitlements were; i) performances, ii) quality of care, iii) patient satisfaction or iv) achieved health.

“You could perhaps reward physicians based on the fact whether health gain was achieved. In this way, you reward the provision of a service, but only if there has been a gain in health. If the patient didn’t benefit from the service, what did you contribute then? I think you shouldn’t be paying something then. Of course it is very difficult to implement a system like this.” [expert]

It goes without saying that a change of entitlement has large implications and an extensive impact on reimbursement policies.

### Question 3: Challenges in the implementation of a more integrated financing mechanism

To generate more knowledge on how financing mechanisms can become less fragmented and more integrated, we asked the respondents what would be challenges in the development of a more integrated financing mechanism for stroke care. The following sub-themes were mentioned by the respondents; *foundations of the financing system, patient population, co-morbidity, clinical pathways, responsibility, differences among stroke services* and *evidence.* Each theme is discussed below.

#### a) Foundations of the financing system

Respondents mentioned that the foundations of the financing system in health care in the Netherlands do not facilitate an integrated financing mechanism. Examples given were that i) there are different macro budgets (e.g. one for primary care and one for hospital care) and a stroke service overlaps these separate budgets and ii) the system of regulated competition does not promote integration of services.

“Everything that you organise in a horizontal fashion, such as stroke services, does not fit into the current system in which we pay for every discipline separately. These are problems which are not so easily solved.” [expert]

In the current healthcare system of regulated competition, healthcare providers as well as insurers are expected to compete with each other. Several stakeholders considered this system to be inappropriate for integrated care;

“Cooperation and regulated competition are in each other’s way. We want the best of both worlds, optimal quality and low cost, and we think that we can achieve that via regulated competition. I think it is safe to say that that is an illusion. Especially in healthcare.” [expert]

#### b) Patient population

Several respondents mentioned that it would be difficult to design one method of payment for stroke care, as the patient population is very diverse.

“There are patients who have almost completely recovered after one week, and there are patients who will be severely handicapped for the next years. It is unclear when is the earliest moment to have a good prognosis for this specific patient, his care trajectory and his needs”. [manager]

Furthermore, there are no objective measures available that indicate the needs of the patient at an early stage. This creates problems between several providers who compete for the same patient, and this also results in situations where patients are placed in suboptimal facilities.

#### c) Co-morbidity

Most of the experts and insurers responded that it is likely that the number of patients with co-morbidity will increase, and separate financing based on a condition might not be optimal for them. For this group of patients, a different type of integrated financing can be more suitable.

“Please don’t divide the patient in 100 conditions. Maybe we end up with chain-care for every chronic disease, and then there will be patients who are in 3 different programs at the same time. We become too specialised in everything, which eventually leads to coordination issues.” [expert]

#### d) Clinical pathways

In addition to differences between patients, the clinical pathways that patients follow can also differ greatly. There are different possible endpoints in the chain of services, and it is unclear when the stroke service as a whole starts and ends. The great number of different care providers who are involved with stroke care, combined with all the possible clinical pathways complicates defining one clear path and attaching a financial figure to this.

“Care for stroke patients is very diffuse care; sometimes there are more than 15 different care providers involved.” [insurer]

#### e) Responsibility and accountability

The issue of responsibility was mentioned several times by respondents. They stated that currently, everyone is responsible for their part of the stroke service instead of the end product, or the overall care for the patient.

“The responsibility of a medical specialist for the total process is limited. As soon as the patient moves on to the next part of the chain, then the physician looses the patient. The money also stops, so there is no incentive to keep track of the patient.” [manager]

When the financing would be more integrated, it is unclear for respondents who should have the final responsibility over the stroke service, and who should receive the money for a patient.

“I think I would only enter such a financial cooperation if I can be the main contractor. I realize that I am saying this out of a fear to get insufficient resources, instead of being confident that we are also a vital part of the chain, and the we will be rewarded accordingly.” [manager]

#### f) Differences among stroke services

The existing stroke services in the Netherlands are in different stages of development, which, according to the respondents, complicates designing one method of payment. Some services have clear agreements and structures and could benefit from an integrated financing mechanism, while for a stroke service that is still in development a simple cooperation fee would be the best option. In addition, not all stroke services have a similar provider structure (e.g. some include only one nursing home, while others have three) and in some regions or in large cities it is more complicated to have clear care chains between fixed providers.

At the same time, some respondents argued that there should remain space for stroke services to organise themselves in the way they want.

“Don’t try to make blueprints for the organisation or financing of the stroke service. I think that it’s OK when they choose to be a loose network instead of a fixed one. But that also doesn’t fit within the existing system.” [expert]

#### g) Evidence

The final point made by respondents was the lack of evidence on the impact of integrated financing on cost-containment. In general, the respondents agreed that improving the financing would result in better quality of care, but respondents were divided in their opinions whether integrated financing lead to more expensive or less expensive care.

## Discussion

Our thematic analysis shows that the current fee-for-service system, according to the respondents, does not provide the right incentives for the integration of stroke care. A number of solutions and different financing models were mentioned by the respondents, but there was no consensus amongst them. Several challenges, related to general factors (e.g. the foundations of the financing system and the issue of co-morbidity), or stroke-specific factors (e.g. diverse patient population and non-uniformity of patient pathways) were mentioned regarding the implementation of a more integrated financing mechanism for stroke care.

### International comparison

The finding that fee-for-service systems do not provide the right incentives for improving quality and efficiency in health care has been recognized by others as well [5,15-17]. In our study, the respondents did not agree amongst each other about the best alternative for the fee-for-service system. Disagreement is also found in the literature. Evidence on pay-for-performance programs for instance seems to depend to a great extent on the specific circumstances and situations of the institutions involved [15,18].

The respondents in our study came up with several suggestions for alternative financing mechanisms for stroke care (presented in Figure 1). There have been financial experiments performed in different groups of chronic patients for several of the proposed payment systems. If we look at those experiments, can we predict the effect of using these systems instead of fee-for-service?

An experiment in Germany for instance, has provided positive evidence for implementation of cooperation fees [19]. In this experiment, sickness funds received higher payments when they set up certified disease management programs and induce patients to enrol while providers were able to receive additional funding if they established integrated care projects. After four years of implementation, a study reported better medical outcomes and significant lower overall costs for diabetes patients enrolled in a disease management program compared to usual care [20].

In the Netherlands, the first experiments with bundled payments for diabetes care have yielded mixed results [21]. Bundled payments in theory rewards providers who have lower costs while penalizing higher-cost providers. In the United States, bundled payments are promoted as the most promising opportunity to control health care spending while encouraging high quality [6,8]. But in accordance with our findings, Davis [17] pointed out that the problem with bundled payments lies in assigning accountability for care across different settings and over time. Care patterns for stroke patients are highly dispersed, there is a lack of continuity in the physician-patient relationship and many different professionals are involved.

Experiments with population-based financing or global payments have been performed in Germany in the Gesundes Kinzigtal experiment [22]. Here, a regional management network together with a physician network and two insurance companies have set up a population-based financing system that is combined with shared-savings. Results so far have shown a reduction in costs of the Kinzigtal region compared to other regions, but future studies have yet to conclude that the decreased costs are indeed due to population health gain. Population-based financing for stroke care in the Netherlands and elsewhere has to be tried out before introduced nationwide. Both results in literature and experiments show mixed effects on the end goal: improving quality of care and reducing costs per patient. The priority now is to experiment with several modes of integrated financing on a small scale, before large national changes will be made.

### Policy implications

Despite the challenges and problems mentioned by the participants, there have been significant improvements in the organisation of stroke care in the Netherlands in the last ten years, without any financial innovations [11]. These developments however have not occurred in every stroke service to the same extent, and also not as fast as is thought necessary to deal with the financial constraints and the rapidly aging population. Therefore, it is important now to move beyond care innovations and to look into the options for financial reforms.

### Strengths and limitations

The strengths of our study include the purposive sampling strategy from different stakeholder groups; in this way we ensured that multiple perspectives were captured through in-depth interviews of highly knowledgeable informants from the five stakeholder groups. The sample included those directly involved in integrated stroke care, and those who were knowledgeable about, but not directly involved in the day-to-day work of stroke services. Anonymisation ensured that respondents felt free to share their own personal opinions. The semi-structured interview technique allowed issues to be explored in a flexible manner. Also, respondents were free to raise any issue that they felt were relevant to the topic under investigation. As a result, it is believed that the information gathered was reflective of genuine concerns and views. Since the respondents were not familiar with the interviewer, we believe that the potential influence of the researchers on data collection is kept minimal. All researchers were involved in analysis and interpretation of the data to ensure that the conclusions accurately reflect the collected opinions and views of the participants.

The main limitation is that we did not include caregivers in the group of patient respondents. Inclusion of this group could have provided additional information. Another limitation could be that the research is performed in the Dutch context, and therefore less applicable to other countries. However, we believe that the Dutch case could be interesting for others because the Dutch system is a fragmented fee-for-service system, which is found in many other countries. Most of the results of this research are specifically applicable to integrated stroke care. However, many of the issues raised regarding stroke care are also applicable for financial and organisational issues in other types of integrated care.

## Conclusions

This study has provided new knowledge on stakeholder perception of the effect of payment systems and financial incentives on cooperation processes, quality of care and cost-containment in integrated stroke care. According to our findings, the current fee-for-service system does not provide the right incentives for the integration of stroke care. It is now necessary to experiment with the different models of financing for integrated stroke care, and report proper cost-effectiveness analyses of the experiments. Depending on the results, nationwide implementation of the optimal models may take place.

## Appendix. Topic guide semi-structured interviews

### 

– Short introduction by interviewer

– Short introduction of interviewee including:

  – Connection to stroke care

  – (If applicable) short description of the integrated stroke chain interviewee is involved in

  – Personal or institutional opinion on integrated (stroke) care

  – Connection to financing of healthcare

### Current financing system

– Impact on quality of stroke care

– Impact on content of stroke care

– Issues with current financing system

– Possible solutions

### Ideal situation

– How do you think integrated (stroke) care should be financed? (probe for detailed answers)

– What should ideal care for stroke patients look like

– Areas for improvement within the existing integrated stroke care

– What are the areas in which improvements can be made specifically regarding quality and efficiency of integrated stroke care?

### Cooperation

– How can/should cooperation be promoted within stroke care

– Is there a role for financing/incentives

### If applicable

– Agreements/deals made between care institutions and with healthcare insurers regarding quality/quantity/efficiency of care

– Managerial perspective: what are the current issues regarding stroke care and the financing of stroke care

– Should healthcare insurers be involved in the development and financing of integrated stroke care, and if so, what should be their task?

– Ministry of Healthcare: experiences with other integrated care programs.

## Competing interests

The authors declare that they have no competing interests.

## Authors’ contributions

All authors contributed to the study’s conceptualization and the study design. JT collected and analyzed the data and prepared the framework of this manuscript. AS and AV led revisions of the paper and provided input into the content of the manuscript. All authors read and approved the final manuscript.

## Pre-publication history

The pre-publication history for this paper can be accessed here:

http://www.biomedcentral.com/1472-6963/13/127/prepub
